# Embedding community-engaged research principles in implementation science: The implementation science center for cancer control equity

**DOI:** 10.1017/cts.2023.32

**Published:** 2023-03-10

**Authors:** Gina R. Kruse, Rebekka M. Lee, Kelly A. Aschbrenner, James G. Daly, Susan Dargon-Hart, Madeline E. Davies, Daniel A. Gundersen, Leslie Pelton-Cairns, Jonathan P. Winickoff, Elsie Taveras, Karen M. Emmons, The ISCCCE Consortium

**Affiliations:** 1 Division of General Internal Medicine, Massachusetts General Hospital, Boston, MA, USA; 2 Department of Social and Behavioral Sciences, Harvard. T. H. Chan School of Public Health, Boston, MA, USA; 3 Department of Psychiatry, Geisel School of Medicine at Dartmouth, Lebanon, NH, USA; The Dartmouth Institute for Health Policy and Clinical Practice, Lebanon, NH, USA; 4 Massachusetts League of Community Health Centers, Boston, MA, USA; 5 Massachusetts General Hospital Kraft Center for Community Health, Boston, MA, USA; 6 Dana Farber Cancer Institute, Cancer Care Delivery Research, Boston, MA, USA; 7 Division of General Academic Pediatrics, Mssachusetts General Hospital, Boston, MA, USA; 8 Mass General Brigham Health, Somerville, MA, USA

**Keywords:** Community engagement, implementation science, health equity, cancer control, community health centers

## Abstract

Gaps in the implementation of effective interventions impact nearly all cancer prevention and control strategies in the US including Massachusetts. To close these implementation gaps, evidence-based interventions must be rapidly and equitably implemented in settings serving racially, ethnically, socioeconomically, and geographically diverse populations. This paper provides a brief overview of The Implementation Science Center for Cancer Control Equity (ISCCCE) and describes how we have operationalized our commitment to a robust community-engaged center that aims to close these gaps. We describe how ISCCCE is organized and how the principles of community-engaged research are embedded across the center. Principles of community engagement have been operationalized across all components of ISCCCE. We have intentionally integrated these principles throughout all structures and processes and have developed evaluation strategies to assess whether the quality of our partnerships reflects the principles. ISCCCE is a comprehensive community-engaged infrastructure for studying efficient, pragmatic, and equity-focused implementation and adaptation strategies for cancer prevention in historically and currently disadvantaged communities with built-in methods to evaluate the quality of community engagement. This engaged research center is designed to maximize the impact and relevance of implementation research on cancer control in community health centers.

## Introduction

The full potential of evidence-based cancer prevention and control interventions has not been realized, particularly in low-income, historically and currently disadvantaged communities [1]. Gaps in the implementation of effective interventions are apparent in nearly all cancer prevention strategies and, in some cases, differences in implementation and the neighborhood or outer context account substantially for disparities in screening outcomes [2]. Federally qualified community health centers (CHCs) are a major source of health care in the US. They serve an estimated 30 million patients, including more than one in five uninsured people and one in five Medicaid recipients [3]. CHCs deliver high-quality care, performing as well as or better than non-CHC primary care practices on a range of quality measures [4,5].

In Massachusetts, despite the excellent environment for preventive care, cancer is the leading cause of death among residents [6] and a source of inequities, with disparities in incidence and outcomes by geographic region, race, ethnicity, and income [7]. Massachusetts CHCs serve more than one million patients in the state, 87% of whom are below 200% of the federal poverty level, 67% identify as racial or ethnic minority, and 37% are best served in a language other than English [8]. Massachusetts CHCs are poised to reduce cancer disparities, but key implementation science innovations are needed to support CHCs in doing so, including 1) effective, low-burden approaches to implementation that minimize burden and workflow disruption; 2) proactive approaches to address equity in implementation planning and execution; and 3) development of sustainable community-engaged partnerships to support ongoing implementation efforts.

The goal of this paper is to provide a brief overview of the Implementation Science Center for Cancer Control Equity, which aims to advance these innovations, and describe how the Center operationalizes its commitment to robust community engagement.

## Methods

### Overview of Partnership Structure

**Fig. 1.**
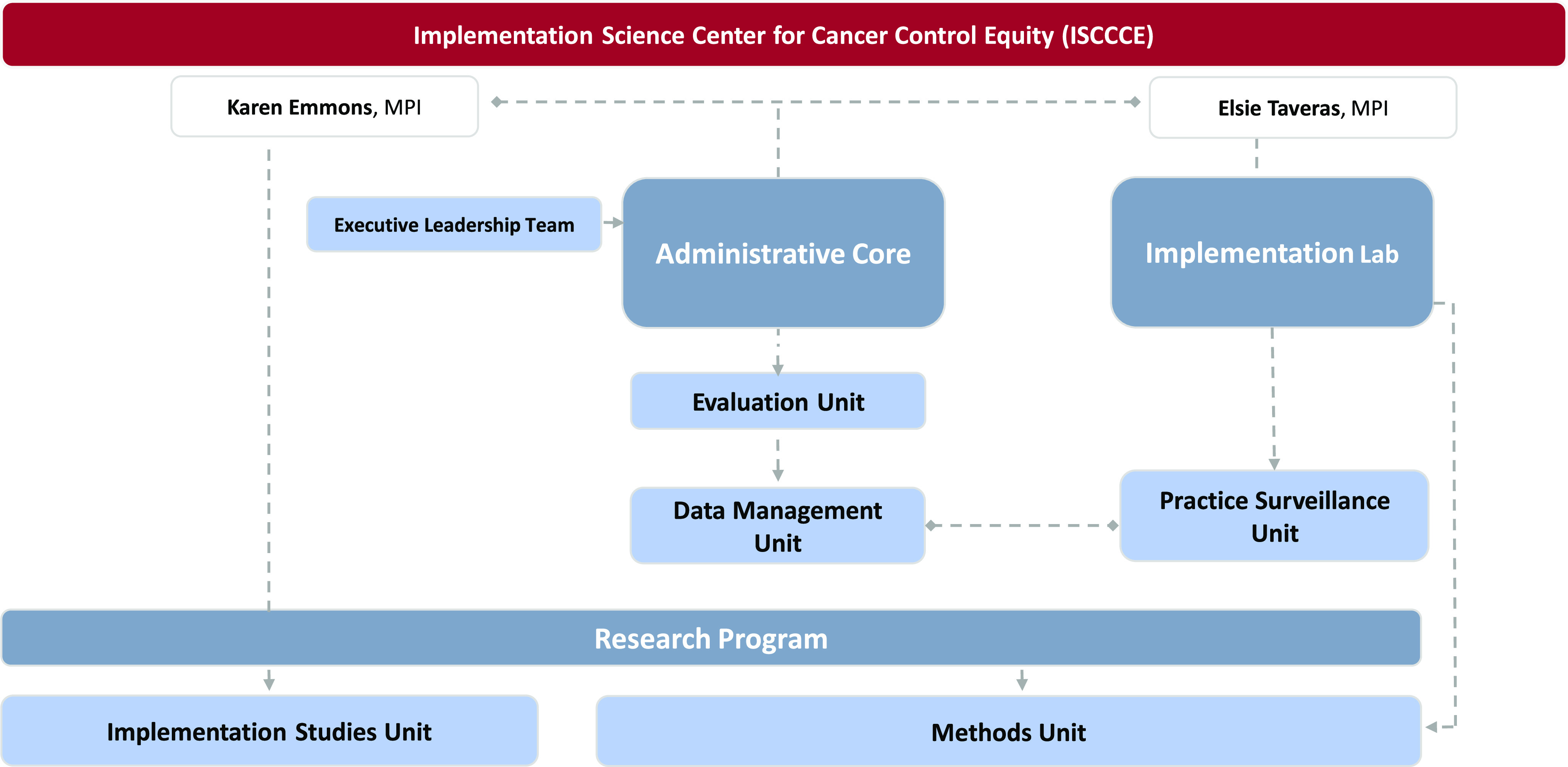
Implementation Science Center for Cancer Control Equity (ISCCCE) organizational structure.

The Implementation Science Center for Cancer Control Equity (ISCCCE, P50 CA244433-01) was funded by the National Cancer Institute as one of seven Implementation Science Centers nationwide. ISCCCE is a partnership among the Harvard T.H. Chan School of Public Health, the Kraft Center for Community Health at Massachusetts General Hospital, and the Massachusetts League of Community Health Centers (MLCHC). We have three specific aims as follows: (1) create an implementation science (IS) ecosystem that engages CHCs in deploying evidence-based interventions (EBIs) for cancer prevention and control; (2) address health inequities by race, ethnicity, income, language, and geography through use of robust IS approaches; and (3) create capacity for addressing ongoing methodologic challenges. ISCCCE is organized with three main components that support a community-engaged infrastructure and an equity-focused research program (Fig. 1).

The core ISCCCE structure includes (1) the Administrative Core, provides governance, and coordinates activities across the center, other University resources, and NCI’s Implementation Science Centers in Cancer Control (ISC^3^) network, and leads data coordination and evaluation; (2) the Implementation Laboratory (I-Lab) builds research capacity and supports implementation of EBIs in CHCs; and (3) the Research Program conducts pilots and research-supporting activities related to both implementation studies and methods. The center takes a collaborative approach with community partners [9], ensuring bi-directional communication. Twice monthly meetings of the leadership team, which includes investigators and community partners, ensure joint decision-making on an ongoing basis. We have bi-monthly all-team meetings that bring together our partners and the broader investigator community. Input through our joint leadership activities is combined with collaborative discussion with CHC staff during quarterly Implementation Learning Community (ILC) meetings. Partner organizations from outside the healthcare system (e.g., health departments, schools, community development groups) are also included in pilots that focus on clinical-community linkages [9,10].

### Community-Engaged Research Principles

All ISCCCE structures and activities have been developed using best practice principles of community-engaged research, as described by the Clinical and Translational Science Awards Consortium’s Community Engagement Key Function Committee Task Force on the Principles of Community Engagement (2011) [11]. The Principles provide evidence-based and practical guidance for engaging community partners in research and place particular attention on the need to engage communities impacted by health issues. We evaluate our efforts to follow these principles on an ongoing basis.

## Results

### Operationalizing Community-Engaged Research Principles

Table 1 summarizes our efforts to operationalize the Principles of Community Engagement. The Principles reflect the grounding of community engagement in the principles of community organization, including fairness, justice, empowerment, participation, and self-determination.

**Table 1.** Operationalization of community-engaged research principles

Community-Engaged Research Principles	Operationalization in ISCCCE
1. **Be clear** about the purposes or goals of the engagement effort and the populations and/or communities you want to engage	- MLCHC’s role as key I-Lab partner and connector to CHCs was defined prior to grant submission. - Incorporated engagement goals into ISCCE organization and leadership structure.
2. **Become knowledgeable** about the community’s culture, economic conditions, social networks, political and power structures, norms and values, demographic trends, history, and experience with efforts by outside groups to engage it in various programs. Learn about the community’s perceptions of those initiating the engagement activities	- MPI had prior collaboration with MLCHC and understood its structures and priorities. - MPI and several co-investigators have extensive familiarity with CHC communities, populations, Community Health Needs Assessments, and cancer trends. - MLCHC provided active input on proposed work scope and budget. - MLCHC provides ongoing information about operating conditions of CHCs, priorities, and competing demands, concurrent initiatives, and opportunities to engage with CHC staff and leadership to develop first-hand knowledge and relationships. - MLCHC and the I-Lab Implementation Learning Community provide opportunities for bi-directional learning and feedback on what is working well and what needs reconsideration. - Investigators learn directly from CHC partners about their context and resources, in the process of partnering in pilot studies.
3. Go to the community, **establish relationships, build trust,** work with the formal and informal leadership, and seek commitment from community organizations and leaders to create processes for mobilizing the community	- Trust was built through active engagement from start of grant writing & budget development. - Input on resources for CHCs suggested significant funding be provided, which was included in budget, with tradeoffs to the science team. - Partnership built on foundation of transparency (budget, titles, resources, etc.) and responsiveness (MPIs prioritized responding to partner queries, emails). - Partners’ input on how to support and engage CHCs was integrated into infrastructure and study design. - Offering flexibility and asking questions that seek to understand CHC strengths and resource constraints, acknowledge CHC challenges, and build trust. - MLCHC is a trusted partner that engages CHC leadership & staff in ISCCCE activities.
4. Remember and accept that collective self-determination is the responsibility and right of all people in a community. No external entity should assume it can bestow on a community the power to act in its own self-interest **(Community empowers itself)**	- Partner CHCs determine if they wish to participate; an open call for all opportunities ensures self-selection and access. - MLCHC provides input on all study ideas, and we do not move projects forward that they do not think are viable or relevant to the CHCs.
5. **Partnering with the community is necessary** to create change and improve health	- MLCHC is actively engaged in all decisions about pilot funding, and research activities. - MLCHC helps drive the implementation activities and ensures efficient integration into the workflow. - During implementation, the I-Lab’s partnership with CHCs may extend to modifications outside of the core activities, where scientifically appropriate and when aligned with CHC health goals. - Research projects have been designed to help CHCs gather needed information from their community partners, and to develop strategies to move that knowledge into action.
6. All aspects of community engagement must **recognize and respect the diversity of the community**. Awareness of the various cultures of a community and other factors affecting diversity must be paramount in planning, designing, and implementing approaches to engaging a community	- We embrace diversity on many levels. From disciplinary perspective, we center our community partner’s input wherever possible, and have brought our MLCHC partners into the leadership team and into leadership (MPI) roles. - Leadership and staff in the CHCs often reflect the racial/ethnic diversity of the communities that they serve, which builds our understanding and awareness of community culture and needs. - CHC partners work with investigators to determine how core elements of interventions or implementation strategies can be modified or adapted to achieve fidelity while optimizing the fit in their context.
7. Community engagement can only be sustained **by identifying and mobilizing community assets and strengths and by developing the community’s capacity** and resources to make decisions and take action	- Most ISCCCE pilots use a population management platform that is shared by the 32 participating CHCs. This allows us to build strategies and tools that can be sustained. - We have created mechanisms for capacity building related to implementation and two-way engagement with the study team. - We actively engage partners in grant writing, presentations and manuscripts to build their capacity and to enrich our understanding of the findings. - We provide mentoring and access to national training opportunities for CHC staff that wish to expand their research skills. - We engage partners as co-investigators on studies where there is interest.
8. Organizations that wish to engage a community as well as individuals seeking to effect change must be prepared to **release control of actions or interventions to the community and be flexible enough to meet its changing needs**	- Our practice surveillance unit is led by our partners in order to ensure unbiased feedback. - Resources provided to CHCs that are participating in implementation studies are designed to be used as flexibly as possible. - A major part of our work is designing an adaptation process that allows CHCs to make changes to their implementation activities to address their priorities, needs, and resources. - We re-design and modify studies when emerging needs dictate a shift (e.g., the pandemic), as warranted by CHC interest. - We intentionally study adaptation, which allows us to further our understanding of how to address the tension between needed flexibility and scientific rigor. - We used new funding opportunities related to COVID to address a key community need.
9. Community collaboration requires **long-term commitment** by the engaging organization and its partners	- We provide expertise in establishing a research infrastructure to the MLCHC, which is developing its own internal research capacity, leveraging the partnership to expand to areas outside of cancer equity. - We routinely meet with partners to identify new sources of funding and make joint decisions as to which opportunities to pursue.

Adapted from Principles of Community Engagement. 2. Washington: US Department of Health and Human Services, 2011.

Abbreviations: MPI = multiple Principal Investigators, ISCCCE = Implementation Science Center for Cancer Control Equity, CHC = Community health center, I-Lab=Implementation Laboratory, MLCHC = Massachusetts League of Community Health Centers.

*Principle 1: Be clear about the purpose of the engagement.* Our partnership with MLCHC was founded on a history of collaboration, largely focused on children’s health [12–14]. When the opportunity for an effort related to cancer prevention and control became available, we immediately met with MLCHC to design a sustainable research partnership to address cancer equity and build CHC capacity for research. MLCHC had been leading efforts to support increased cancer screening, and it became clear that our interests were aligned and that there was an opportunity for two-way learning. Several of our ISCCCE design features were recommended by our partners, such as the use of a learning community, having MLCHC gather input on CHC staff perceptions of partnership quality, and designing an open and engaged process for identifying pilot research topics.

*Principle 2: Become knowledgeable about the community.* The MPIs had prior collaborations with MLCHC and with communities in which the CHCs are located and had conducted prior research in the CHC setting [12,15–20]. A key part of our structure is having routine contact with our partners so that we not only understood the community context at the outset, but that we were aware of emerging issues that impact the CHCs and their communities. Close communication during the COVID-19 pandemic allowed us to pivot our research activities to ensure that we were respectful of the pressures on the CHCs. We were able to apply for funding through the NIH Rapidly Accelerating Diagnostic Testing for COVID-19 in Underserved Populations (RADx-UP) mechanism, which supported testing expansion in partner CHCs, and allowed us to design implementation research to understand the circumstances that allow CHCs to respond to emerging public health crises [10].

*Principle 3: Establish relationships and build trust.* Despite our existing relationships, ISCCCE significantly ramped up the level of collaboration and engagement. There were new partners introduced into the existing relationships, and we needed to ensure that trust permeated all relationships. We worked to build trust from the very beginning of ISCCCE planning, grant writing and budget development, prioritizing transparency, and receiving active input from MLCHC on the work and the budget. For example, one recommendation from MLCHC was the provision of funding for the CHCs to support their participation in pilot studies. We co-developed principles for determining what level of support was needed and budgeted for these. Support is based on study requirements and typically provides $15,000 to $50,000 to each CHC participating in implementation pilots. In our RADx-UP grant, CHC deliverables were significantly greater than in other pilots, and thus we provided $375,000 per participating CHC over two years.

Our development of an Implementation Learning Community (ILC) has provided opportunities for direct engagement and relationship development and to showcase the efforts of CHC staff in pilots, all of which contribute to the development of trust. The ILC is comprised of quarterly meetings, hosted virtually to allow for broad participation, and online learning materials. Two-hour ILC meetings include ISCCCE progress and updates, presentations by pilot CHCs describing implementation successes and challenges, expert speakers, moderated panels of implementing teams and investigators, and facilitated group discussions to integrate the speaker or panel topics with local realities. A range of staff, from executive leadership to community health workers, are invited to participate. The discussions are co-facilitated by ISCCCE team members and CHC staff. Additional online learning tools include 1) an online learning platform sharing IS resources such as curated resources, ILC recordings, and ISCCCE products and publications; and 2) access to quality improvement coursework. The ILCs are bi-directional and have engaged agendas to provide a venue for building trust and research capacity.

*Principle 4: Community empowers itself.* Our operating practices have been designed to maximize CHC autonomy related to participation in the ISCCCE. For example, we adopted MLCHC’s recommendation for how we approach engaging CHCs in pilot studies, by offering open webinars that are hosted by MLCHC in which study opportunities are explained, and interested parties can ask questions and provide input. MLCHC sends a recording of the webinars to all CHC leaders and manages a streamlined application and review process for interested CHCs. This process enables interested CHCs to choose to participate rather than be repeatedly approached by individual investigators, and MLCHC “pre-vets” opportunities so that CHC leaders know that the MLCHC, their trusted partner, supports the work.

**Fig. 2.**
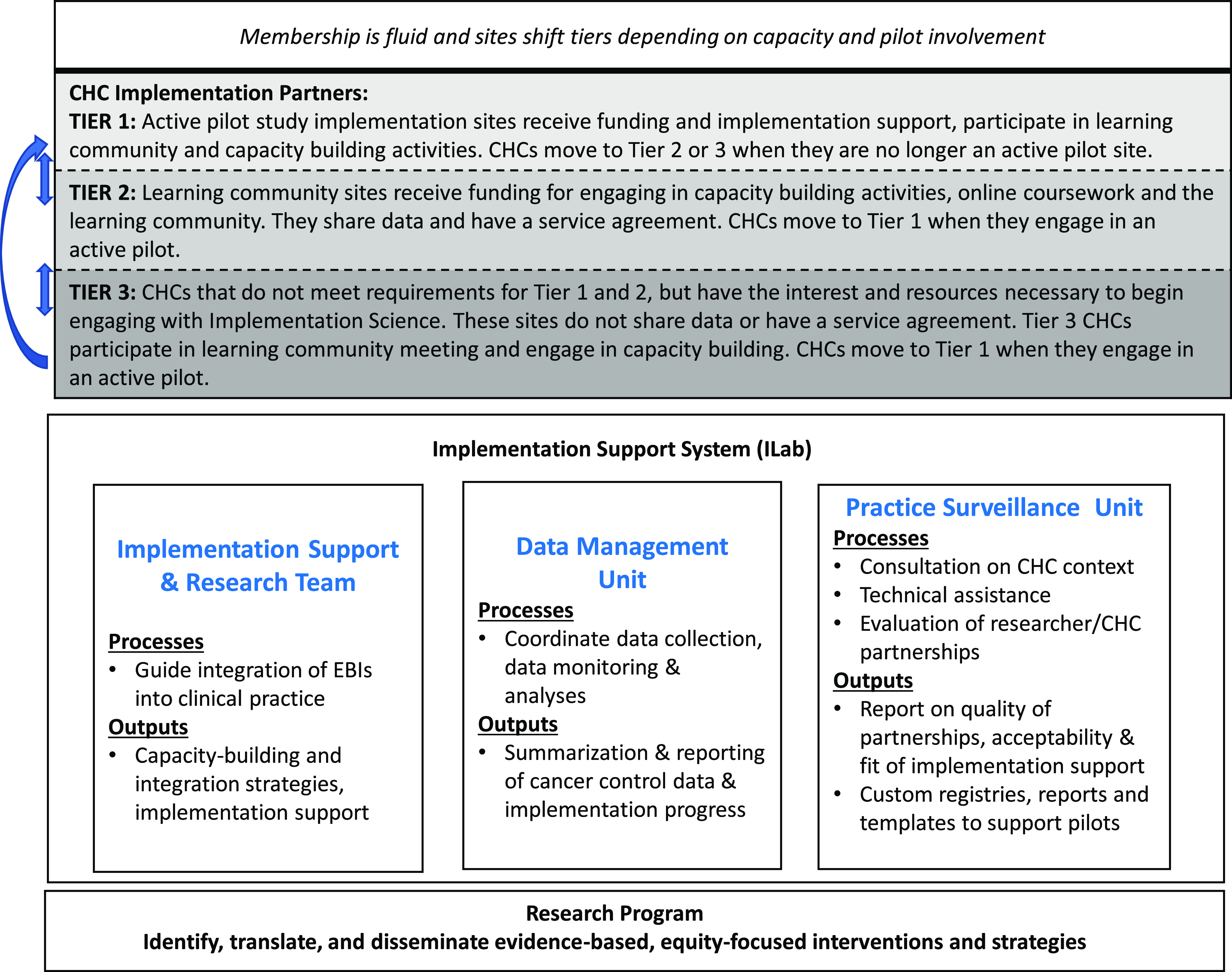
Implementation lab membership and infrastructure. CHC, community health center; EBI, evidence-based interventions.

Our efforts to recognize and build on partner autonomy are also reflected in the structure of the Implementation Laboratory (I-Lab), which uses a tiered membership approach that allows CHCs to engage in implementation research according to their readiness (Fig. 2). Tier 1 CHCs are active pilot sites. Tier 2 CHCs receive financial support to allow staff and leadership to engage with the ILC to build capacity toward participation in the later pilots. Other CHCs that are not ready to actively engage in pilots are invited to participate as they are interested (Tier 3), with the goal of developing research readiness for the future. The tiers are fluid to accommodate changes in CHC interests, readiness, and capacity. For example, the number of Tier 1 CHCs increased from four in Year 1 to 14 in Year 3. Fig. 2 depicts the I-Lab membership and structure.

*Principle 5: Partnering with the community is necessary to create change.* Our organizational structure places MLCHC at the leadership table. They actively engage in all decisions about pilot funding and research activities. Further, MLCHC helps drive all implementation activities; its Vice President of Clinical Health Affairs is a member of the I-Lab and is involved in designing and running ISCCCE implementation pilots. This integration of clinical leadership into the I-Lab has enabled us to enhance our learning on how to leverage quality improvement strategies in the context of IS and to respond more effectively to implementation barriers. Through our ILC, we are able to identify issues that the CHCs are struggling with and to partner directly with them to support action. For example, in RADx-UP, a key issue for the CHCs was to determine how best to partner with local community organizations as they ramped up COVID testing and outreached at-risk groups. In the fall of 2020, we conducted a rapid needs assessment that entailed 84 semi-structured qualitative interviews with 107 community health center staff, community partners, and community residents. We used a two-phase framework approach to analyze the data, including deductive coding to facilitate rapid analysis for action so that the CHCs could incorporate the learning into their testing strategy, followed later by an in-depth thematic analysis. The CHCs reported finding this approach extremely helpful in designing their testing outreach [9].

*Principle 6: All aspects of community engagement must recognize and respect the diversity of the community.* There are many aspects to diversity in the context of community-engaged research. First, there is disciplinary diversity, in that as scientists we must respect and honor the tacit and operational knowledge that community partners provide. We center MLCHC’s input through our leadership and operating structures. Second, there is contextual diversity, in that the resources and needs of each CHC are unique, and thus, a one-size fits all approach to engagement does not work. With MLCHC’s recommendation, we use a deliverables approach to budgeting with the CHCs, providing considerable flexibility in how they use the resources offered. We work with investigators to specify what elements of an implementation strategy or intervention require high fidelity and these are specified as deliverables. Then, we work with CHC partners on how core elements can be modified or adapted to achieve fidelity while optimizing the fit in their context. For example, a pilot study measuring an implementation strategy that bundled outreach for Fecal Immunochemical Testing (FIT) with screening for social needs was designed to have core functions and flexible forms in which those functions could be operationalized. The core fidelity elements included outreach to offer bundled screening and follow-up to promote FIT return. CHCs had the flexibility to choose how they followed up (e.g., by telephone, text, or letter) and who they prioritized for outreach according to their staffing models or programmatic priorities.

We also seek out opportunities to learn directly from CHC staff. Staff in the CHCs reflect the racial/ethnic diversity of the communities they serve, and many staff are residents of these same communities. We prioritize our learning directly from CHC staff to ensure that we build our understanding and awareness of community culture and needs.

We also aim to contribute to the diversity of the scientific community by creating an inclusive environment to support interns, students, postdocs, and junior faculty to advance their career development in community-engaged, equity-focused implementation research. The I-Lab hosts underrepresented student summer interns. We have career development opportunities for postdoctoral fellows and junior faculty who are underrepresented in medicine. The I-Lab also prioritizes hiring research staff from the communities we partner with. Inclusion of diverse staff and trainees allowed us to have broad language representation in the community needs assessment conducted for our RADx-UP project; interviews with community residents were conducted in English, Spanish, Vietnamese, and Arabic, reflecting populations served by participating CHCs.

*Principle 7: Community engagement is sustained by identifying and mobilizing community assets and strengths and by developing community capacity and resources.* We have benefitted significantly from the FQHC’s use of the population management and reporting platform, *Data Reporting and Visualization System* (DRVS), that enables them to submit required data to HRSA, and also provides extensive tools to track progress toward required quality measures. Thirty-two CHCs in MA utilize this EHR-agnostic system, which allows a systematic and standardized way to collect outcome data. We have prioritized interventions that can be implemented through the platform. The partnerships have applied for funding to interventions in DRVS that address cancer prevention and control needs and can be integrated into CHC workflows.

We actively invest in the human resources at our partner CHCs. For example, we supported participation of a CHC-based researcher and epidemiologist as a co-investigator in multiple research projects. We provide ongoing mentoring, as well as financial support for CHC researcher participation in a national IS training program. We have made community partner participation in publications and presentations a requirement for ISCCCE investigators, and several senior leaders at the CHCs have highlighted the value of this opportunity for their staff [9,10,21–23]. Partner contributions have enriched our ability to interpret and act on our findings, while building CHC capacity.

Our evaluation unit benefits from MLCHC’s support for data mapping, accuracy, and training. The research team has learned from the strategies that MLCHC uses to ensure data quality. We believe the benefits have been bi-directional; in that, our analysis of CHC data expands the way our partners typically approach their data, adding new knowledge and understanding of factors influencing outcomes. The evaluation team has developed a data ecosystem using REDCap [24,25], a HIPAA-compliant data platform, that allows us to efficiently capture, store, and link multiple data types (e.g., qualitative, quantitative, administrative) from different sources (e.g., surveys, EHRs) over time, and, importantly, that enable us to rapidly access data to answer new and emerging questions using individual-, contextual-, and system-level data. Moreover, the upfront resource investment in designing and implementing such a data ecosystem lessens the burden on community partners to extract and review data on a case-by-case or project-by-project basis.

*Principle 8: Community engagement requires a release of control of actions or interventions to the community and the flexibility to meet its changing needs.* Our ongoing practice and partnership surveillance is overseen by our MLCHC partners, who are closest to the CHC’s work and in the best position to gather unbiased input from the CHCs. To provide feedback on our efforts to operationalize effective and equitable partnerships, MLCHC gathers input from CHC staff who have participated in pilot studies by administering an online survey that aims to understand their experience with the implementation research process (e.g., clarity and reasonableness of the requirements, experience carrying out the work) and partnership quality (Supplement Materials 1). MLCHC shares summaries of this data to inform improvements in partnership methods.

Summary data from CHC partners in our initial implementation pilot highlighted 1) needed improvements in I-Lab online coursework and the need for more robust onboarding when new CHC staff joined ongoing pilots; and 2) positive experiences with research partnerships such as the research requirements being clearly communicated and reasonable, the feasibility of implementation activities within regular health center workflows, and CHC staff feeling supported to deliver interventions and respected by the ISCCCE team for their knowledge and contributions.

Release of control to partners is also accomplished by including flexibility in our collaborations. For example, we were about to launch a pilot study in early 2020 focused on bundling breast and colorectal cancer screening when the pandemic hit. CHCs were inundated with COVID-related activities, but also trying to figure out how to deliver home-based care where possible and to address patient’s social needs. Driven by CHC priorities, the pilot was pivoted to study FIT testing, a home-based cancer screening strategy, and social needs screening and referral, as described above. In addition, a major activity within ISCCCE is the development of an adaptation process that supports CHCs to make changes to their implementation activities to address their local priorities, needs, and resources [21]. Developing dynamic implementation and adaptation strategies to fit emerging implementation challenges and evolving workflows increases the likelihood of sustainment [26].

*Principle 9: Community collaboration requires long-term commitment.* We entered the partnership with MLCHC with the goal of developing a lasting infrastructure to support implementation of evidence-based cancer prevention and control interventions. We are working towards this goal by responding to as many funding opportunities that are of interest to our partners as possible and by actively engaging trainees in this work so that we can ensure a pipeline of investigators who are well-trained and committed to community-partnered research. We are also actively collaborating with MLCHC in the development of their own research infrastructure, The Health Equity Research and Policy Institute, which will support their long-term engagement in research. The ISCCCE MPIs serve on the search committee for the Institute Director and provide support to develop the Institute’s research infrastructure.

## Discussion

ISCCCE aims to grow capacity for implementation research among the federally qualified health centers in Massachusetts and to produce equity-focused implementation tools for dissemination to CHCs across the country. At the core of our work is the belief that it will be built on strong community partnerships, center community needs and goals, and build on community assets. In this paper, we have illustrated our efforts to embed the principles of community engagement in all that we do, from our structure to our budget, our pilot study selection, and throughout all our processes with the intent of sharing our experience in operationalizing these principles with others interested in community engagement.

From the very beginning of the development of our vision, the center has considered best practice principles of community engagement. We built on existing trusted relationships and were intentional about how to integrate new team members. Embedded feedback mechanisms are designed to maximize the likelihood of honest and objective feedback. This provides opportunities for course correction and to amplify methods that are working well. We were intentional about building in the flexibility to continue to improve our methods for partner engagement and capacity building with the guidance and direction of our partners.

We have found in prior work that it is relatively straightforward to integrate some of the Principles of Community-Engaged Research into our research endeavors, but much harder to attend to all of them, as we have attempted to do in ISCCCE. Adherence to these multiple principles has, at times, resulted in the need to modify the pace of research progress as we have pivoted due to partner needs and priorities. However, our work is better adapted to our partner’s environment, more nuanced, relevant, and sustainable as a result.

The practice and research partners in ISCCCE have largely non-overlapping resources and networks, and by leveraging all of them, rather than just those of the research team, we are building on and expanding these investments. We are able to bring in new innovations that sit both within and outside of the research sphere. This produces bi-directional leveraging and bi-directional learning. We anticipate that our center will contribute to the growing literature measuring the effects of community engagement on the impact and outcomes of partnered research [27]. Community-engaged implementation research takes a committed investment of time and resources, but we anticipate that applying this approach will maximize the returns both in terms of the science produced and its real-world sustained impact on closing cancer equity gaps in communities.

## Data Availability

Data generated through center activities will be shared in accordance with Cancer Moonshot funding policies.

## Supplementary material

For supplementary material accompanying this paper visit https://doi.org/10.1017/cts.2023.32. click here to view supplementary material
