# Supplementary material for: Embedding community-engaged research principles in implementation science: The implementation science center for cancer control equity
Source: J Clin Transl Sci. 2023 Mar 10;7(1):e82. doi: 10.1017/cts.2023.32 (PMC10130842; doi:10.1017/cts.2023.32)
Supplement: Supplementary file 1 [file S2059866123000328sup001.docx]

**Practice Surveillance Survey for ISCCCE Pilot CHCs**

The League is working with the Harvard ISCCCE team to better understand your experience participating in the Dual Screening research study. One of the objectives of this project is to build satisfying, productive bi-lateral partnerships between the researchers and health center teams. The information that you provide the League through this survey will help to flag if course corrections need to be made during the pilot. You will receive this survey again at the close of the pilot to gain a full understanding of your experience over the course of the project.

1. I work at:
   1. CHC A
   2. CHC B
   3. CHC C
   4. CHC D

**Understanding of Grant Requirements**

**For this grant, I am clear about the following requirements as outlined in the scope of work:**

**Strongly Disagree/Agree/Neutral/Disagree/Strongly Disagree**

1. Data collection requirements
2. Attendance at Implementation Learning Community meetings
3. Participation in meetings with the ISCCCE Implementation Team coaching health centers to implement the intervention.
4. Participation in the virtual learning platform, CANVAS. (not the IHI membership)

Optional Comments:

The following requirements were reasonable to carry out at our health center.

1. Data collection requirements
2. Attendance at Implementation Learning Community meetings
3. Participation in meetings with the ISCCCE Implementation Team coaching health centers to implement the intervention
4. Participation in the virtual learning platform, CANVAS

**Experience of Relationship and Carrying Out Work**

**Please tell us the extent to which you agree or disagree with the following statements:**

**Strongly Agree/Agree/Neutral/Disagree/Strongly Disagree**

1. I feel that the research outcomes will benefit our health center and patients.
2. The dual screening intervention and protocols to be tested are clear to me.
3. My team felt included in the shaping of the intervention.
4. I feel respected by the ISCCCE team for my knowledge and contributions.
5. We are able to incorporate the dual screening intervention into our regular health center workflows.
6. I feel supported by the ISCCCE team to effectively deliver the dual screening intervention.
7. The ISCCCE implementation team was sensitive to the constraints and realities of my health center.
8. Adaptations to the dual screening intervention have helped us to deliver a better intervention for our patients.
9. The ISCCCE team was sensitive to the needs of our patient population.
10. The intervention will be sustainable beyond this grant.
11. I would be enthusiastic about participating in another research study with the ISCCCE team.
12. The resources provided by the grant are sufficient to support our participation in the dual screening pilot.

Optional comments:

**Usefulness of Supports Provided**

**How useful have the following supports been to you in implementing the intervention, or other evidence-based practices?**

**N/A-Have NOT participated in this support/To a great extent/Somewhat/Very Little/Have participated, but not at all useful**

1. Meetings with the ISCCCE Implementation Team coaching health centers to implement the intervention.
2. Learning Community Meetings that include other health centers
3. Virtual Learning Platform – CANVAS
4. IHI Open School Membership
5. Training to better utilize DRVS
6. The use of DRVS tools

Optional comments:

**Open-ended questions**

1. What factors helped your CHC to implement this research project?
2. What barriers did you face in implementing this research project, whether internal (e.g. lab interface) or external factors (e.g. reimbursement)?
3. Please include any other comments you have about participating in the ISCCCE Dual Screening Project.
4. Please list any topics that you would prioritize for the next set of pilots?
